# Clinical management of ageing people living with HIV in Europe: the view of the care providers

**DOI:** 10.1007/s15010-020-01406-7

**Published:** 2020-03-20

**Authors:** Marta Boffito, Lene Ryom, Christoph Spinner, Esteban Martinez, Georg Behrens, Jürgen Rockstroh, Johannes Hohenauer, Karine Lacombe, Mina Psichogyiou, Norbert Voith, Patrick Mallon, Teresa Branco, Veronica Svedhem, Antonella dÁrminio Monforte

**Affiliations:** 1 Chelsea and Westminster Hospital NHS Foundation Trust, London, UK; 2 Rigshospitalet University of Copenhagen, Copenhagen, Denmark; 3 Technical University of Munich, School of Medicine, Munich, Germany; 4 Hospital Clinic, Barcelona, Spain; 5 Medical University Hanover, Hanover, Germany; 6 University of Bonn, Bonn, Germany; 7 BDO Health Care Consultancy, Vienna, Austria; 8 Sorbonne University Hospital St Antoine, Paris, France; 9 National and Kapodistrian University of Athens, Athen, Greece; 10 Option 3, Vienna, Austria; 11 UCD School of Medicine, Dublin, Ireland; 12 Hospital Prof. Doutor Fernando Fonseca, Amadora, Portugal; 13 Karolinska University Hospital, Stockholm, Sweden; 14 ASST Santi Paolo E Carlo University Hospital, Milan, Italy

**Keywords:** HIV, Ageing, Care delivery, Delphi method, Europe

## Abstract

**Background:**

Although guidelines for the management of HIV infection include recommendations for aging people living with HIV (PLWH), clinical practice of European HIV care providers may vary.

**Method:**

We performed a study using a 3-phase Delphi methodology by involving a panel of clinicians with expertise in HIV infection clinical management. The main aim of the study was to assess the care provider prospective on how HIV clinical care should be delivered to ageing PLWH. The first phase involved ten clinicians to identify HIV comorbidities of interest. The second and third phases recruited clinicians virtually via a web-based questionnaire that included 137 questions focussed on 11 comorbidities (e.g. cardiovascular disease, pulmonary disease, etc.).

**Results:**

Results were analysed thematically and consensus (or not) among European physicians reported. Ninety-seven and 85 responses were collected in phase 2 and 3, respectively. High levels of agreement were found among clinical care providers across Europe and with the European AIDS Conference Society guidelines regarding key items of clinical management of comorbidities in ageing PLWH.

**Conclusion:**

However, we identified some important gaps, such as the lack of standardisation or implementation of the assessment of frailty or menopause, which are emerging as important factors to optimise ageing PLWH clinical care. Further studies are warranted to confirm whether intensified screening translates into HIV morbidity advances.

**Electronic supplementary material:**

The online version of this article (10.1007/s15010-020-01406-7) contains supplementary material, which is available to authorized users.

## Introduction

The introduction of combination antiretroviral therapy (cART) in the mid-1990s changed HIV from a rapidly deteriorating condition associated with significant mortality to a complex, chronic disease, in which people living with HIV (PLWH) have now and increase in life expectancy to “near normal” levels. Consequently, the demographics and clinical characteristics of PLWH have changed dramatically, with a growing proportion of individuals aged more than 60 years [1–3].

There is evidence that PLWH have an increased risk of several non-AIDS comorbidities [4, 5] with the most common comorbidities among PLWH, including cardiovascular disease, renal and liver impairment, cancer and neurocognitive impairment [6]. The increased risk of comorbidities among PLWH may be a consequence of (1) immune dysregulation caused by the HIV infection, (2) accumulating antiretroviral drug-related toxicity, (3) lifestyle factors and (4) co-infections [7]. Importantly, this represents unique challenges for PLWH, health care providers, wider society, and strategies to deliver optimal individualised clinical care to this population are needed to prevent and manage the risk of mortality, drug-drug interactions and polypharmacy.

Several international organisations, such as the European AIDS Clinical Society (EACS) and US Department of Health and Human Services (DHHS), provide extensive guidelines and recommendations on how to screen for and manage comorbidities in PLWH [8–11]. Despite this, the actual clinical practice of European HIV care providers and whether guidelines are implemented merit systematic investigation. Indeed, research suggests that despite the growing number of guidelines, their use in practice is frequently reported as being unpredictable and complex [12, 13]. Given the importance of the implementation of guidelines, we performed a Delphi study, which involved a panel of HIV experts, based in Europe to investigate the clinical management of ageing PLWH from the perspective of the care provider.

## Methods

### Study design

**Fig. 1:**
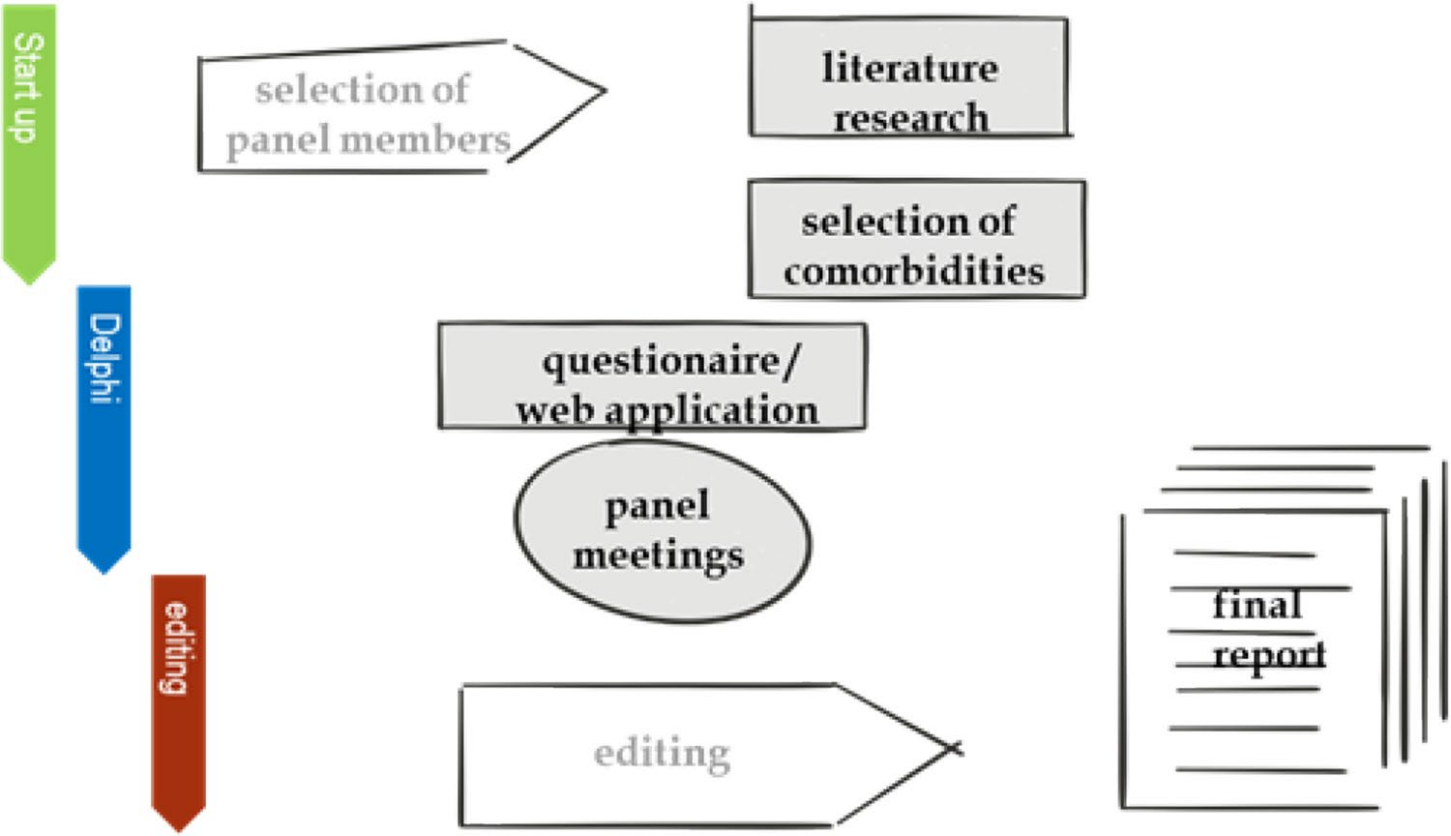
Delphi methodology

A Delphi technique [14, 15] was used to identify research topic priorities in HIV clinical care management. Beyond the present study, the Delphi methodology has been used previously in the field of HIV management [16–18]. The Delphi technique is an iterative research method used to seek consensus and refine the input of a group of experts on a subject in a systematic manner [19], and can appropriately serve as an alternative to a committee meeting or task force. To this end, the Delphi technique utilises a series of well-defined questionnaires based on surveys and feedback (Fig. 1).

### Procedure and sample

**Fig. 2:**
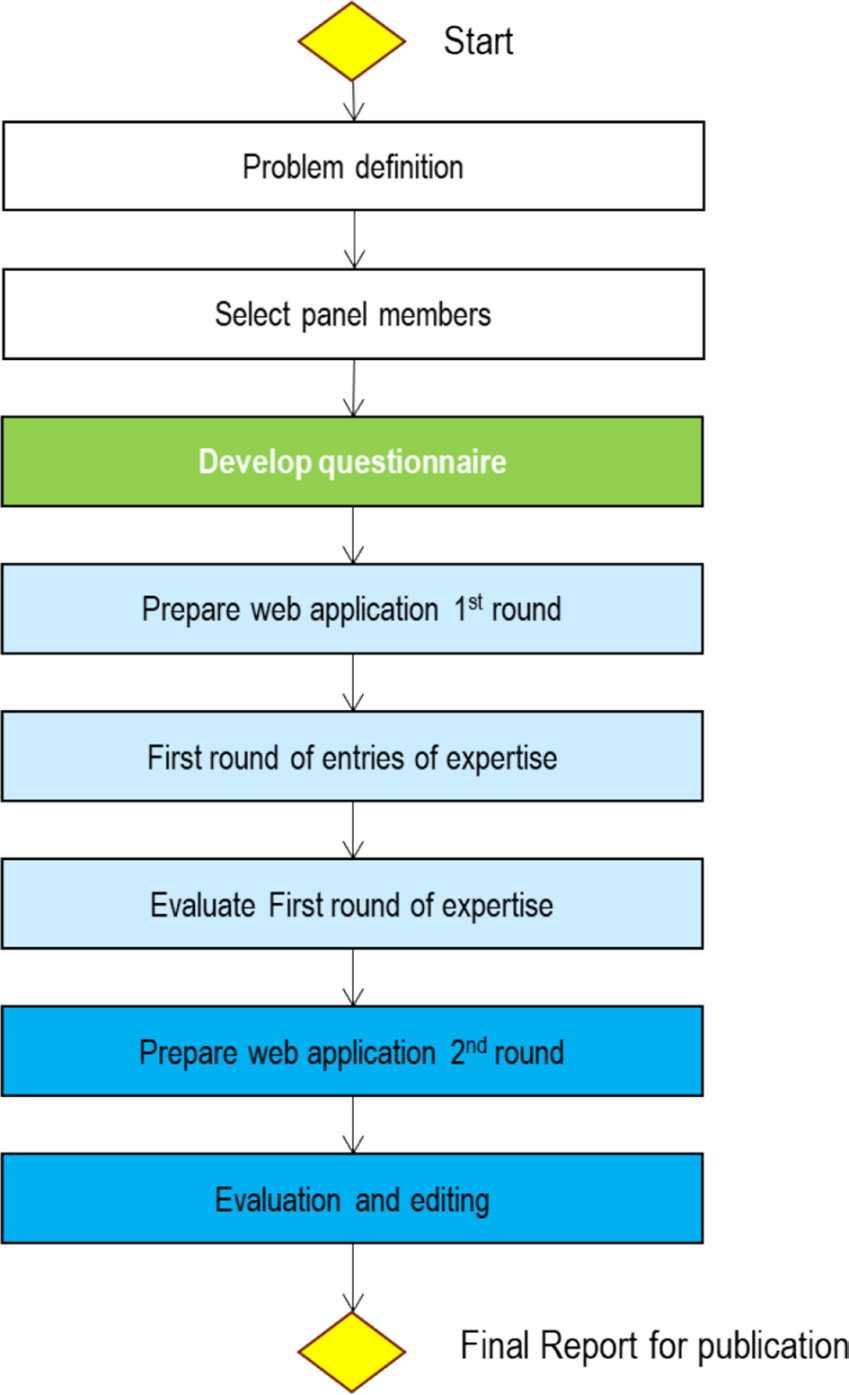
Delphi process

The present study used a 3-phase Delphi methodology (Fig. 2). In the first phase, ten experts were identified; the experts were all clinicians involved in HIV clinical care, from Denmark, France, Germany, Greece, Italy, Portugal, Romania, Spain, Sweden and the United Kingdom, who were able to dedicate time to the project. The ten experts met twice in two different European cities (in Milano on October 9th, 2018 and in Vienna on February 12th, 2019) with the objective of identifying and agreeing on priority comorbidities as a focus for the subsequent phases, as well as score ranges to help with data interpretation. The result of this consensus was the base for a web-based Delphi questionnaire that focused on HIV clinical management. The questionnaire comprised 137 items across 11 central comorbidities, including cardiovascular disease (such as hypertension and peripheral vascular disease), pulmonary disease, metabolic disorders (including diabetes and dyslipidaemia), liver impairment, kidney impairment, urogenital disorders, bone disorders (such as osteoporosis), symptoms related to the central and peripheral nervous system, mental health, sexual and reproductive health, cancer, and other infectious diseases and vaccination. For each item, relevance was measured on a scale of 0–9 (with 0 representing “not relevant” and 9 representing “highest relevance”), and the frequency for which the item should be checked was assessed (possible responses were 3–4 months, 6 months, 12 months, 2–5 years, the same as for the general population, and never). Additionally, there was the possibility to differentiate the answers by patient groups: age group, gender, HIV-acquisition risk and immunological status.

In the second phase, we invited HIV physicians and/or specialist nurses (in countries where nurses run HIV outpatient services independently and in parallel with physician-led clinics, e.g. Sweden and UK), who were nominated by our ten experts, to complete a Delphi survey, using the web-based database questionnaire developed in the first phase. The scoring was done via a multiple-choice web application hosted at www.epid.at based on a Microsoft SQL-Server. In the third phase, the ten experts evaluated the responses to the web-based questionnaire from the participants and further developed the questionnaire to incorporate any feedback. If agreement on an item was reached in the second phase, the item was not included in the next version of the questionnaire. Patient groups which received 25% or more responses were included in the revised questionnaire and those receiving less than 25% were omitted. Subsequently, the revised web-based questionnaire was sent to the participants to reassess their decisions according to the Delphi method, based on the results of the scoring of all participants in the previous round.

### Analysis of data

All responses to the web-based questionnaire were analysed and summarised thematically. The consensus result was the score which received the most responses (i.e. a plurality) from the responding participants. Median relevance was calculated from the responders who deemed the item as relevant (i.e. it excludes those who selected “not relevant”).

## Results

### Responders

**Fig. 3:**
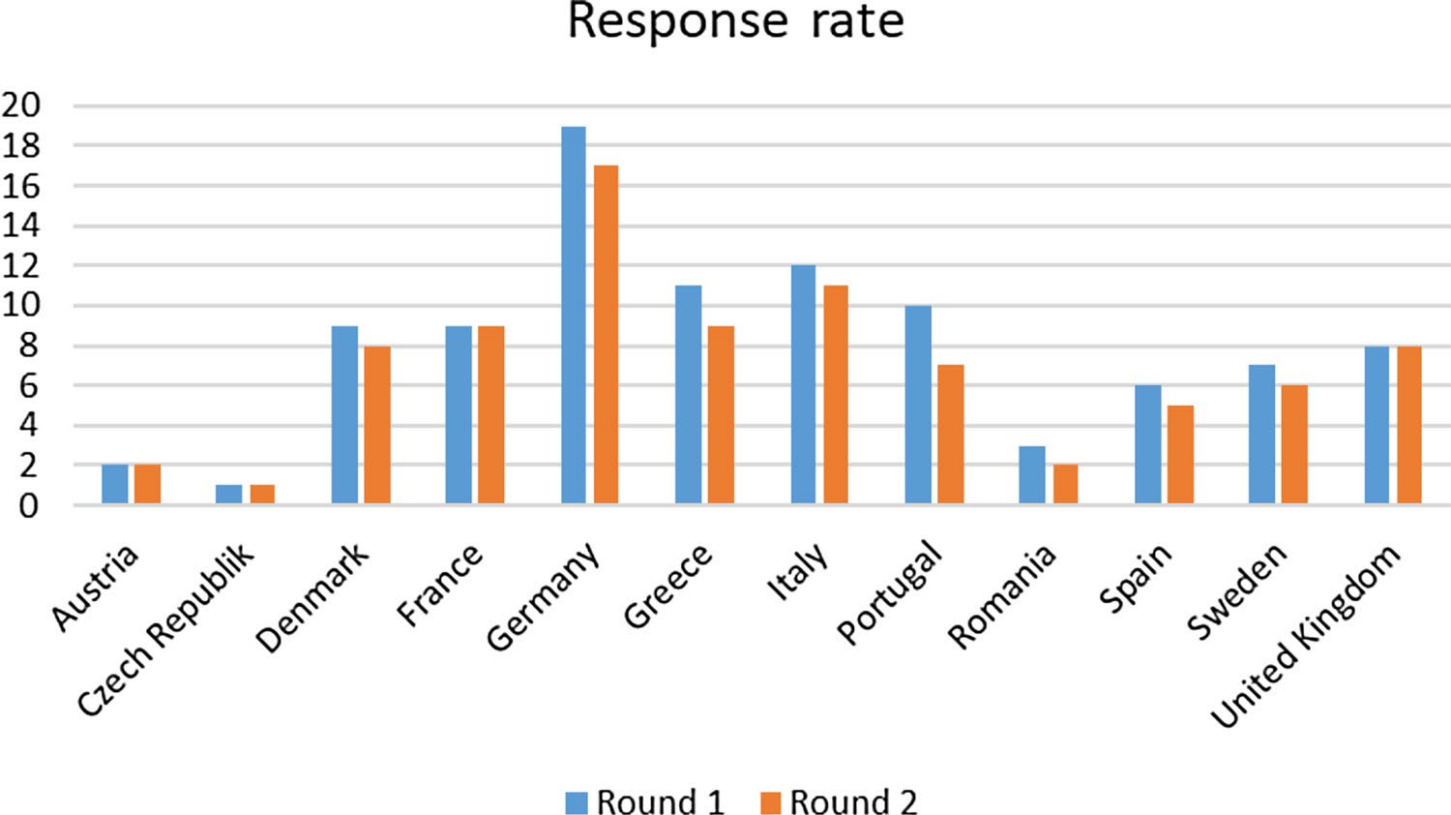
Geographical distributions of the responses of the questionnaire

HIV physicians and/or specialist nurses were invited to participate in the first round of the web-based questionnaire and 97 responded. Of these, 85 also participated in the final round by completing the revised web-based questionnaire. The geographical distribution of the responders is illustrated in Fig. 3. The greatest proportions of responders were from Germany (*n* = 19, 19.6%), Italy (*n* = 12, 12,4%) and Greece (*n* = 11, 11.3%).

### Relevance and frequency of items

**Fig. 4:**
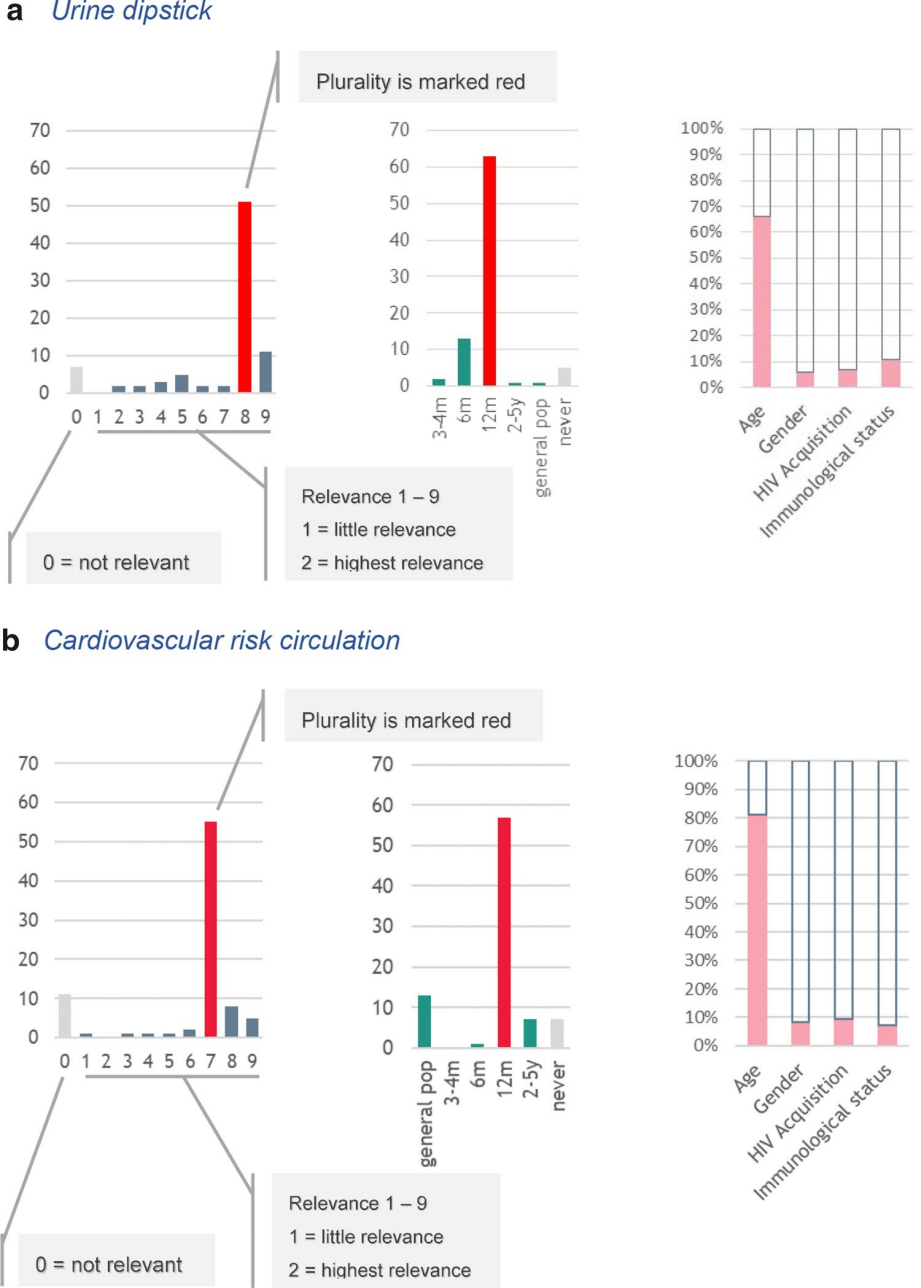

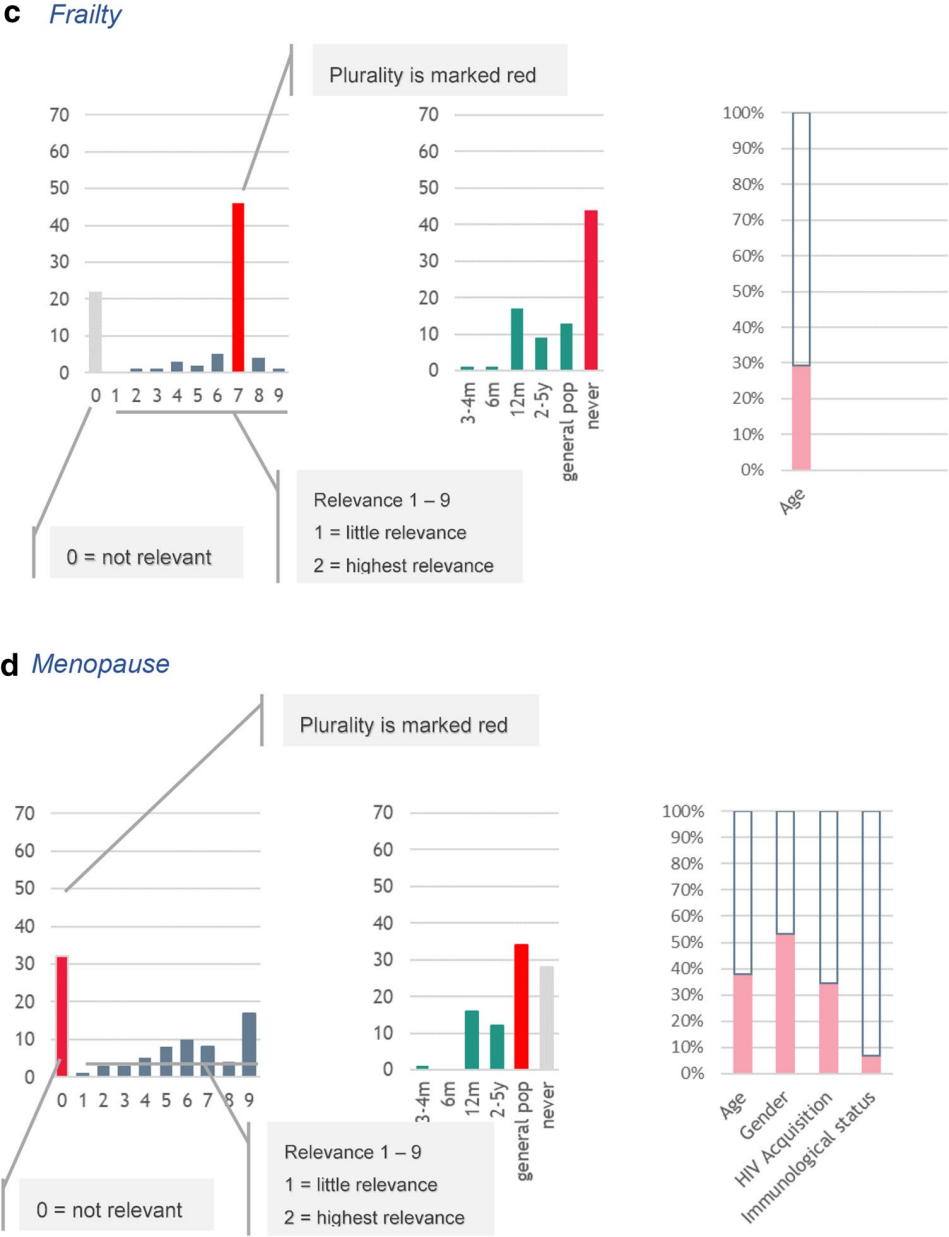
Examples of the distribution of results: **a** urine dipstick; **b** cardiovascular risk calculation; **c** frailty; **d** menopause

**Fig. 5:**
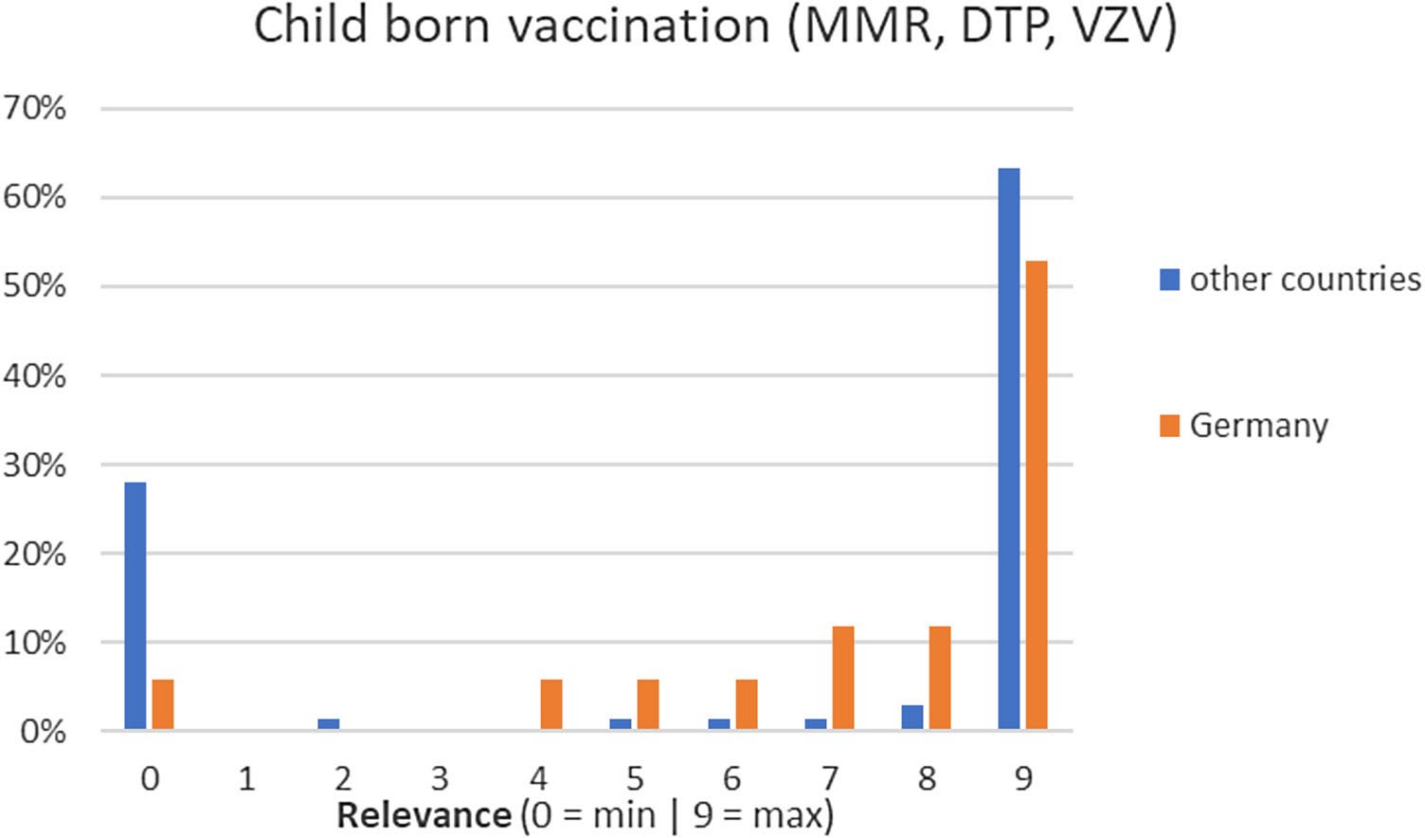
Perceived relevance of childborn vaccination

Annex 1 summarises the responders’ plurality relevance scores, as well as the frequency of assessment for the comorbidity items included in the web-based questionnaire (Figs. 4, 5).

#### Cardiovascular disease (CVD, including hypertension and peripheral vascular disease)

There was general agreement in the relevance of performing the Framingham score or other risk assessment tools such as the DAD (Data Collection on Adverse Events of Anti-HIV Drugs) CVD risk score (plurality relevance = 7/9), with responders predominantly selecting a frequency of once every 12 months. Responders considered assessment of blood pressure (8/9), renal impairment (9/9) and heart rate (7/9) to be relevant, at a frequency of every 6 months. Stress electrocardiogram (ECG) (0/9), echocardiogram (0/9) and carotid Doppler assessment (0/9) were considered by responders as not relevant and to be performed at the same frequency as in the general population. Finally, other assessments such as the Q-risk Assessment Score (0/9), Coronary Artery Calcium Score (0/9), homocysteine (0/9), walking distance test (0/9), New York Heart Association (NYHA) scale (0/9) and Ankle Brachial Index (0/9) were considered as not relevant by the majority of responders and were reported as never assessed.

#### Pulmonary disease

Responders considered assessment of smoking (9/9) as highly relevant, at a frequency of every 12 months, and assessment of allergy history (9/9) professional environment (7/9), chest X-ray (7/9) and respiratory rate (7/9) at a similar frequency as in the general population to be relevant; however, in PLWH, these last two assessment would not performed as screening tools in the absence of any symptomatology. Assessment of exposure to allergens in a, spiroergometry (0/9), arterial blood gas analysis (0/9) and echocardiogram of the right side of the heart (0/9) were considered by responders as not relevant and were all assessed at a similar frequency as in the general population. Tuberculosis investigation was considered as a relevant item according to HIV acquisition groups (9/9). Finally, assessment of pulse (0/9), and chest computed tomography (CT) (0/9) scan were considered by responders as not relevant and were reported as never performed.

#### Metabolic disorders

Assessment of triglyceride (8/9), total cholesterol (8/9), high-density lipoprotein (HDL) (9/9) and low-density lipoprotein (LDL) (9/9) were all considered by responders as highly relevant, and assessed at a frequency of every 12 months. Responders considered fasting glucose (8/9) as relevant and to be assessed every 6 months. There was high agreement among responders that oral glucose tolerance test (0/9) fasting insulin (0/9), Homeostasis Model Assessment (HOMA) of insulin (0/9), C-peptide (0/9), and apolipoproteins A and B (0/9) were not relevant and these assessments were never predominantly assessed. Furthermore, responders considered assessment of fasting glucose (8/9) to be slightly more relevant than glycated haemoglobin (HbA1c) (7/9).

Responders also agreed that Body Mass Index (BMI) (8/9) should be measured every 12 months, but waist circumference was less relevant (7/9), but was never assessed. Responders noted that frailty should be assessed in individuals with advanced age (7/9), at a frequency of every 12 months, but frailty score (0/9) was considered as not relevant.

#### Kidney impairment

There was agreement among responders in the relevance of assessing most of the renal markers. In particular, responders considered assessment of serum electrolytes (potassium and sodium) (8/9), serum creatinine (9/9), serum creatinine-based estimated clearance, using the Cockroft–Gault equation (8/9) and estimated glomerular filtration rate by the chronic kidney disease epidemiology collaboration (CKD-EPI) equation or others (id est the MDRD equation) (8/9) as relevant and to be performed every 6 months, while urine dipstick (8/9) and albumin-to-creatinine ratio in urine (6/9) were to be performed every 12 months. Responders considered assessment of ultrasound (0/9), as well as urine albumin and protein over 24 h (0/9), as not relevant.

#### Liver impairment

Assessment of alanine transaminase (ALT)/aspartate transaminase (AST) (9/9), total bilirubin (9/9), alkaline phosphatase (ALP) (9/9) and platelets (8/9) were considered by responders as highly relevant, and were assessed every 6 months, while hepatitis virus markers (9/9) were also highly relevant and assessed every 12 months. Responders considered liver ultrasound (7/9), non-invasive fibrosis assessment (6/9) rather relevant and to be performed as in general population. Elastography assessment was considered as non-relevant (0/9).

#### Bone disorders

High levels of agreement were reached by responders for bone disorders. The fracture risk assessment tool (FRAX), with or without dual-energy X-ray absorptiometry (Dexa), was considered by responders as relevant (6/9) and was assessed for female (7/9) every 2–5 years and yearly in older population (8/9). Assessment of vitamin-D levels in serum was considered as relevant (7/9) and was assessed every 12 months. Blood markers of bone damage such as serum calcium (7/9) and phosphorus (7/9) were considered by responders to be assessed every 12 months. Responders considered assessment of parathyroid hormone (5/9) as a screening tool less relevant, and hip and spine X-ray (0/9) as not relevant, and these items were never assessed.

#### Central and peripheral nervous system

Responders identified age as an important differentiator for performing neurocognitive assessment tests (6/9), which were performed at the same frequency as in the general population. There were high level agreement among responders for all other assessments [fundoscopy, lumbar puncture, CT scan, magnetic resonance imaging (MRI), electroencephalography (EEG), electromyography], which were all considered as not relevant (0/9).

#### Mental health

Screening for depression and anxiety was rated as an important tool that needs to be repeated yearly (7/9). Responders considered assessing tobacco addiction (8/9) as relevant, as measured by determining the number of packs per year. Assessment of alcohol addiction was not considered by responders as relevant and was predominantly never assessed, while assessment of recreational drug addiction was performed only in specific settings of HIV acquisition.

#### Sexual and reproductive health

There was high agreement among responders that assessment of sexually transmitted infections (such as syphilis, chlamydia, gonorrhoea, viral hepatitis) (9/9), sexual dysfunction (7/9) and contraception in women with HIV (9/9) should be performed every 12 months. Responders considered investigating menopause and use of oral contraceptives in women as not relevant (0/9).

#### Cancer

The majority of screening tests for cancer were recommended to be performed at the same frequency as in the general population. Responders considered performing a cervical Papanicolaou smear in women living with HIV with annual interval as relevant (9/9). On the other hand, performing an anal Papanicolaou smear in men who have sex with men (MSM) was considered as relevant (7/9), but responders reported that they do not perform it routinely.

#### Other infectious diseases and vaccinations

Responders considered vaccinations of hepatitis A and B (9/9), human papilloma virus (HPV) (8/9), child born diseases (9/9), diphtheria, tetanus and pertussis (9/9), as well as assessment of toxoplasmosis antibodies (9/9) and cytomegalovirus (CMV) serology (9/9), as relevant and all to be performed at the same frequency in all ages without a particular focus on older patients. Responders also considered influenza vaccination as highly relevant, and to administered annually. Meningococcal vaccination, varicella–zoster virus (VZV) vaccination, tuberculin skin test or interferon gamma release assay, CMV polymerase chain reaction and assessment of Cryptococcus antigen were all predominantly considered by responders as not relevant.

## Discussion

Our study showed that the Delphi method was useful in reaching consensus among international experts regarding important items relating to the clinical management of ageing PLWH. By applying this method, we were able to develop a questionnaire designed to assess the level of agreement (or disagreement) in the management of key items in PLWH among clinical care providers.

We found high levels of alignment among clinical care providers across Europe and the recommendations of the EACS, suggesting that clinical guidelines are implemented in clinical practice. However, we observed a number of conditions which have been shown by recent literature to be important for ageing PLWH, but seemingly still remain to be assessed uniformly in clinical care. Emerging evidence from the current literature underlines the importance of frailty assessment in ageing PLWH [20]. However, the results of our study suggest that clinicians do not routinely assess frailty using an appropriate score; this observation may be primarily due to the lack of a validated score in PLWH. Interestingly, while there was generally high agreement on regular screening for sexually transmitted infections and sexual dysfunction, menopause assessment was not considered of high relevance in women living with HIV [21]. Evidence for the impact of HIV infection on the age of onset of menopause is thus far conflicting, and additional studies that assess the impact of HIV on cART response are required. Indeed, the use of hormonal substitution therapy may increase risk of drug–drug interactions, which is important not to overlook [10].

We observed a discrepancy between how clinicians consider vitamin D assessment should be performed and recommendations from international guidelines. Participants in our study considered that vitamin D status should be assessed on a yearly basis: However, studies of vitamin D use for fracture prevention in PLWH have been inconsistent [22] and guidelines recommend to measure vitamin D only at HIV presentation and in PLWH who have low bone mineral density and/or high risk for fracture [10]. Responders reported that FRAX, with or without Dexa scan, should be calculated every 2–5 years, which aligns with the recommendation from the EACS [10].

There was high agreement among responders for the routine assessment of liver and renal function tests, urine dipstick, and cervical smear in women, which is in line with current recommendations from the EACS [10]. There was a lack of agreement among responders in terms of performing anal HPV screening and Papanicolaou smear cytology in MSM; this observation probably reflects the controversial nature of these assessments and that they have not been shown to provide a benefit on early rectal cancer identification [10]. Furthermore, anal smears are often not performed because of lack of resources or expertise, which may not be available in all countries where the questionnaire has been administered.

Responders reported that fasting glucose was the preferred method of assessment for diabetes screening, which differs from guideline recommendations in the general population [23]. In PLWH, the EACS guidelines recommend using either fasting glucose, HbA1c or glucose tolerance testing for diabetes screening [10]. This observation may be related to concerns about use of Hb1Ac in PLWH, specifically relating to its precision under certain circumstances (i.e. severe liver or kidney impairment) or the availability of the test for routine monitoring across Europe.

While our study showed agreement on the value of assessing depression and anxiety annually, the absence of standardised neurocognitive testing is likely due to the unavailability of a practical and validated standardised neurocognitive test in PLWH. Furthermore, available specialised testing is time consuming and requires considerable training. However, given the high prevalence of neurocognitive impairment in the ageing cohort of PLWH, neurocognitive testing is important in those with relevant risk factors or who present with relevant symptoms [10].

We observed variability among responders regarding the relevance and frequency of vaccinations in PLWH. These observations likely reflect the different immunisation practices by European country [24].

The limitations of our study are those that usually characterise questionnaire-based research [25]. A major limitation relates to the complexity and size of the questionnaire itself, which may have led to responder fatigue for the latter part of the questions. Finally, while this study considered the opinions of HIV experts who designed the questionnaire and the HIV specialists who responded, it did not involve PLWH being in care. However, there are other initiatives that focus on the patient perspectives, such as the British HIV Association (BHIVA) standards and the EmERGE initiative.

In conclusion, our study highlighted high levels of agreement regarding key items of clinical management of comorbidities in PLWH among HIV care providers across different European countries and with international guidelines. However, our study identified several clinically important gaps in the clinical care of ageing PLWH, such as the need for standardised assessment of frailty and menopause. These findings may increase the awareness for the need to optimise and further standardise clinical care delivery to ageing PLWH, who are at risk of comorbidities, polypharmacy and drug–drug interactions. Further studies are needed to assess if such intensified systematic screening translates into improvement in morbidity and mortality among PLWH.

## Author contributor

AW: Imperial College London. ASt: Ifi Institute. AB: APHP. ACM: Centro Hospitalar de Lisboa Ocidental-Hospital de Egas Moniz-Serviço de Infecciologia e Medicina Tropical. AT: Karolinska Univ Hospital. AM: Klinikum rechts der Isar. AS: University of Bari-A.O.U. Policlinico Bari. AP: University General Hospital “Attikon” Medical School–Sational and Kapodistrian University of Athens. AI: Bellvitge University Hospital. BR: Azienda Ospedaliera Universitaria Senese. BMC: Unit of Infectious Diseases ARNAS Garibaldi Catania. BS: IIMK. CJT: South general hospital infec dis clin. CP: INMI Spallanzni. CW: MVZ Karlsplatz. CM: SEGAS. CJ: Klinikum Rechts der Isar. CS: University Hospital Munich. CS: Klinikum rechts der Isar, München. CC: Region Västmanland. CL: Karolinska Sjukhuset. CB: Universität Bonn. CL: University hospital cologne. CG: ASST FBF Sacco. DP: Risghospitalet, University of Copenhagen CHIP. DR: Papa Giovanni XXIII. DS: APHP. ES: General Hospital of Nikea and Piraeus. EE: Chelsea and Westminster Hospital. EB: COREVIH pays de la loire. EM: Hospital Clínic de Barcelona. EV: Praxis am Ebertplatz, Cologne. ET: Hospital dos Capuchos. FB: Clinic of Infectious Diseases, San Paolo Hospital, University of Milan, Italy. FA: Praxiszentrum Alte Mälzerei. GB: Hannover Medical School. GK: Risghospitalet, University of Copenhagen CHIP. GB: Venhälsan, Infektion, South Hospital. GM: Chelsea and Westminster Hospital. HN: Infectious Disease Dept, University Hospital of Skane, 221 85 Lund. HB: Uppsala University Hospital. II: Victor Babes Hospital Bucharest. JG: Bichat University Hospital Paris. JM: University Hospital of Cologne. JR: Chelsea Westminster Hospital. JS: Klinikum rechts der Isar. JC: CNWL. JV: Barreiro Hospital. JR: Universität Bonn. KR: Private practice. KG: Chelsea Westminster NHS trust. KD: Risghospitalet, University of Copenhagen CHIP. KP: ATTIKON University Hospital. LW: CNWL. LS: S.Anna Hospital. LSK: ID unit, Dept. of Internal Medicine, University Hospital of Zeeland, Roskilde. LR: CHIP. LH: Chelsea and Westminster Hospital. LC: University Hospital of Martinique. LMC: Hospital Clinic Barcelona. LL: Patras university Hospital. MC: Imperial College London. MA: Infectious Diseases Clinic Hospital Galati. MF: Centro Hospitalar Universitario do Algarve. MCM: Asst Niguarda. MP: ational and Kapodistrian University of Athens. MH: Bordeaux University Hospital. MA: G.Gennimatas hospital athens. NV: CHU Saint Antoine. NF: Hospital dos Marmeleiros. NS: Infectious Diseases Unit, ASST-MONZA, San Gerardo Hospital, Monza. NM: Hospital Garcia de Orta. PL: Sotiria Hospital. PP: 251 Hellenic airforce general hospital. PS: AP-HP. RCA: ULS Matosinhos, EPE-Hospital Pedro Hispano. RV: Mannheimer Onkologie Praxis. RS: Centro Hospitalar s João. SJ: Aarhus University Hospital. SN: Klinikum rechts der Isar. SN: Ospedale San Raffaele. SK: Andreas syggros" hospital. SDN: University of copenhagen rigshospitalet. TB: Centro Hospitalar de Lisboa occidental. TB: Hvidovre Hospital. UK: Praxis Dr. Kastenbauer. VP: Hôpital Pitié-Salpêtrière. VB: Azienda Ospedaliero Universitaria di Modena. VP: Evaggelismos general hospital. VS: Karolinska University Hospital. VA: Hospital univwrsitario central de asturias. VS: Red Cross Hospital, Athens.

## Electronic supplementary material

Below is the link to the electronic supplementary material.

Supplementary file1 (DOCX 78 kb)
