# Correction to: Clinical management of ageing people living with HIV in Europe: the view of the care providers

**DOI:** 10.1007/s15010-020-01463-y

**Published:** 2020-06-24

**Authors:** Marta Boffito, Lene Ryom, Christoph Spinner, Esteban Martinez, Georg Behrens, Jürgen Rockstroh, Johannes Hohenauer, Karine Lacombe, Mina Psichogyiou, Norbert Voith, Patrick Mallon, Teresa Branco, Veronica Svedhem, Antonella dÁrminio Monforte

**Affiliations:** 1 Chelsea and Westminster Hospital NHS Foundation Trust, London, UK; 2 Rigshospitalet University of Copenhagen, Copenhagen, Denmark; 3 Technical University of Munich, School of Medicine, Munich, Germany; 4 Hospital Clinic, Barcelona, Spain; 5 Medical University Hanover, Hanover, Germany; 6 University of Bonn, Bonn, Germany; 7 BDO Health Care Consultancy, Vienna, Austria; 8 Sorbonne University Hospital St Antoine, Paris, France; 9 National and Kapodistrian University of Athens, Athen, Greece; 10 Option 3, Vienna, Austria; 11 UCD School of Medicine, Dublin, Ireland; 12 Hospital Prof. Doutor Fernando Fonseca, Amadora, Portugal; 13 Karolinska University Hospital, Stockholm, Sweden; 14 ASST Santi Paolo E Carlo University Hospital, Milan, Italy

## Correction to: Infection 10.1007/s15010-020-01406-7

The original version of this article unfortunately contained a mistake. The name Lene Ryom was incorrect. The corrected author list is given above.

The original article has been corrected.

