# Supplementary material for: Clinical management of ageing people living with HIV in Europe: the view of the care providers
Source: Infection. 2020 Mar 20;48(4):497–506. doi: 10.1007/s15010-020-01406-7 (PMC7395037; doi:10.1007/s15010-020-01406-7)
Supplement: Supplementary file 1 — Supplementary file1 (DOCX 78 kb) [file 15010_2020_1406_MOESM1_ESM.docx]

**LIST OF AUTHORS AND AFFILIATIONS**

| Name | Institution | City, Country |
| --- | --- | --- |
| Marta Boffito | Chelsea and Westminster Hospital NHS Foundation Trust | London, UK |
| Lene Ryom Nielsen | Rigshospitalet  University of Copenhagen | Copenhagen, Denmark |
| Christoph Spinner | University Hospital Klinikum rechts der Isar, Munich | Munich, Germany |
| Esteban Martinez | Hospital Clinic | Barcelona, Spain |
| Georg Behrens | Medical University Hanover | Hanover, Germany |
| Jürgen Rockstroh | University of Bonn | Bonn, Germany |
| Johannes Hohenauer | BDO Health Care Consultancy | Vienna, Austria |
| Karine Lacombe | Sorbonne University Hospital St Antoine | Paris, France |
| Mina Psichogyiou | National and Kapodistrian University of Athens | Athen, Greece |
| Norbert Voith | Option 3 | Vienna, Austria |
| Patrick Mallon | UCD School of Medicine | Dublin, Ireland |
| Teresa Branco | Institute Prof Teresa Branco | Lisbon, Portugal |
| Veronica Svedhem | Karolinska University Hospital | Stockholm, Sweden |
| Antonella dÁrminio Monforte | ASST Santi Paolo e Carlo University Hospital | Milan, Italy |

**CORRESPONDING AUTHOR:**

Dr. JOHANNES HOHENAUER

BDO Health Care Consultancy GmbH

Karl Popperstr.4, 1100 Vienna, Austria

Tel: +43/1/53737-0

Email: Johannes.Hohenauer@bdo.at

#### Cardiovascular (inclusive hypertension and peripheral vascular)

| **Items screened** | **Relevance** | | | | | | | | | | **Frequency** | | | | | |
| --- | --- | --- | --- | --- | --- | --- | --- | --- | --- | --- | --- | --- | --- | --- | --- | --- |
|  | 0 | 1 | 2 | 3 | 4 | 5 | 6 | 7 | 8 | 9 | 3-4m | 6m | 12m | 2-5y | Gen | never |
| Risk assessment tool: Framingham or other tools such as D.A.D. |  |  |  |  |  |  |  | **󠄃** |  |  |  |  | **●** |  |  |  |
| Pat.grp: <25 |  |  |  |  |  |  | **󠄃** |  |  |  |  |  | **●** |  |  |  |
| Pat.grp: >50 |  |  |  |  |  |  |  |  | **󠄃** |  |  |  | **●** |  |  |  |
| Risk assessment tool: Q-risk | **󠄃** |  |  |  |  |  |  |  |  |  |  |  |  |  |  | **●** |
| Coronary artery calcium score (CACS) | **󠄃** |  |  |  |  |  |  |  |  |  |  |  |  |  |  | **●** |
| Walking distance test | **󠄃** |  |  |  |  |  |  |  |  |  |  |  |  |  |  | **●** |
| New York heart association (NYHA) | **󠄃** |  |  |  |  |  |  |  |  |  |  |  |  |  |  | **●** |
| Heart Rate |  |  |  |  |  |  |  | **󠄃** |  |  |  | **●** |  |  |  |  |
| Electrocardiography (ECG) |  |  |  |  |  |  |  | **󠄃** |  |  |  |  |  |  | **●** |  |
| Pat.grp: <25 |  |  |  |  |  |  | **󠄃** |  |  |  |  |  |  |  | **●** |  |
| Stress electrocardiography (ECG) | **󠄃** |  |  |  |  |  |  |  |  |  |  |  |  |  | **●** |  |
| Homocystein | **󠄃** |  |  |  |  |  |  |  |  |  |  |  |  |  |  | **●** |
| Echocardiogram | **󠄃** |  |  |  |  |  |  |  |  |  |  |  |  |  | **●** |  |
| Carotid Doppler | **󠄃** |  |  |  |  |  |  |  |  |  |  |  |  |  | **●** |  |
| Venous / arterial Doppler | **󠄃** |  |  |  |  |  |  |  |  |  |  |  |  |  | **●** |  |
| Renal impairment |  |  |  |  |  |  |  |  |  | **󠄃** |  | **●** |  |  |  |  |
| Blood pressure |  |  |  |  |  |  |  |  | **󠄃** |  |  | **●** |  |  |  |  |
| Pat.grp: >50 |  |  |  |  |  |  |  |  |  | **󠄃** |  | **●** |  |  |  |  |
| Ankle brachial index (ABI) | **󠄃** |  |  |  |  |  |  |  |  |  |  |  |  |  |  | **●** |

#### Pulmonary disease

| **Items screened** | **Relevance** | | | | | | | | | | **Frequency** | | | | | |
| --- | --- | --- | --- | --- | --- | --- | --- | --- | --- | --- | --- | --- | --- | --- | --- | --- |
|  | 0 | 1 | 2 | 3 | 4 | 5 | 6 | 7 | 8 | 9 | 3-4m | 6m | 12m | 2-5y | Gen | never |
| Tobacco |  |  |  |  |  |  |  |  |  | **󠄃** |  |  | **●** |  |  |  |
| Allergy history |  |  |  |  |  |  |  |  |  | **󠄃** |  |  |  |  | **●** |  |
| Tuberculosis (TB) |  |  |  |  |  |  |  |  |  | **󠄃** |  |  |  |  | **●** |  |
| Pat.grp: Heterosexual |  |  |  |  |  |  |  |  | **󠄃** |  |  |  |  |  | **●** |  |
| Pat.grp: MSM |  |  |  |  |  |  |  |  | **󠄃** |  |  |  |  |  | **●** |  |
| Professional exposure |  |  |  |  |  |  |  | **󠄃** |  |  |  |  |  |  | **●** |  |
| Pulsimetry | **󠄃** |  |  |  |  |  |  |  |  |  |  |  |  |  |  | **●** |
| Respiratory frequency |  |  |  |  |  |  |  | **󠄃** |  |  |  |  |  |  |  | **●** |
| Chest X-ray |  |  |  |  |  |  |  | **󠄃** |  |  |  |  |  |  | **●** |  |
| Pat.grp: >50 |  |  |  |  |  |  |  |  | **󠄃** |  |  |  |  |  | **●** |  |
| Pat.grp: HP-Country |  |  |  |  |  |  |  |  | **󠄃** |  |  |  |  |  | **●** |  |
| Pat.grp: IVDU |  |  |  |  |  |  |  |  | **󠄃** |  |  |  |  |  | **●** |  |
| Spirometry | **󠄃** |  |  |  |  |  |  |  |  |  |  |  |  |  | **●** |  |
| Arterial blood gas analysis | **󠄃** |  |  |  |  |  |  |  |  |  |  |  |  |  | **●** |  |
| Computed tomography (CT) scan | **󠄃** |  |  |  |  |  |  |  |  |  |  |  |  |  |  | **●** |
| Echocardiography (right heart) | **󠄃** |  |  |  |  |  |  |  |  |  |  |  |  |  | **●** |  |

#### Metabolic disorders (incl. diabetes and dyslipidaemia)

| **Items screened** | **Relevance** | | | | | | | | | | **Frequency** | | | | | |
| --- | --- | --- | --- | --- | --- | --- | --- | --- | --- | --- | --- | --- | --- | --- | --- | --- |
|  | 0 | 1 | 2 | 3 | 4 | 5 | 6 | 7 | 8 | 9 | 3-4m | 6m | 12m | 2-5y | Gen | never |
| Nutrition (healthy diet) |  |  |  |  |  |  |  | **󠄃** |  |  |  |  | **●** |  |  |  |
| Frailty |  |  |  |  |  |  |  | **󠄃** |  |  |  |  |  |  |  | **●** |
| Frailty score | **󠄃** |  |  |  |  |  |  |  |  |  |  |  |  |  |  | **●** |
| Body mass index (BMI) |  |  |  |  |  |  |  |  | **󠄃** |  |  |  | **●** |  |  |  |
| Waist circumference |  |  |  |  |  |  |  | **󠄃** |  |  |  |  |  |  |  | **●** |
| Pat.grp: <25 |  |  |  |  |  |  | **󠄃** |  |  |  |  |  |  |  |  | **●** |
| HbA1c |  |  |  |  |  |  |  | **󠄃** |  |  |  |  |  |  | **●** |  |
| Fasting glucose |  |  |  |  |  |  |  |  | **󠄃** |  |  | **●** |  |  |  |  |
| Random glucose |  |  |  |  |  |  |  | **󠄃** |  |  |  |  |  |  |  | **●** |
| Triglyceride |  |  |  |  |  |  |  |  | **󠄃** |  |  |  | **●** |  |  |  |
| Oral glucose tolerance test (OGTT) | **󠄃** |  |  |  |  |  |  |  |  |  |  |  |  |  | **●** |  |
| Fasting insulin | **󠄃** |  |  |  |  |  |  |  |  |  |  |  |  |  |  | **●** |
| Estimation of insulin sensitivity: HOMA | **󠄃** |  |  |  |  |  |  |  |  |  |  |  |  |  |  | **●** |
| Estimation of insulin sensitivity: QUICKI index | **󠄃** |  |  |  |  |  |  |  |  |  |  |  |  |  |  | **●** |
| C peptide | **󠄃** |  |  |  |  |  |  |  |  |  |  |  |  |  |  | **●** |
| Total cholesterol |  |  |  |  |  |  |  |  | **󠄃** |  |  |  | **●** |  |  |  |
| High density lipoprotein (HDL) |  |  |  |  |  |  |  |  |  | **󠄃** |  |  | **●** |  |  |  |
| Pat.grp: <25 |  |  |  |  |  |  |  |  | **󠄃** |  |  |  | **●** |  |  |  |
| Low density lipoprotein (LDL) |  |  |  |  |  |  |  |  |  | **󠄃** |  |  | **●** |  |  |  |
| Pat.grp: <25 |  |  |  |  |  |  |  |  | **󠄃** |  |  |  | **●** |  |  |  |
| Thyroid stimulating hormone (TSH) |  |  |  |  |  |  | **󠄃** |  |  |  |  |  |  |  | **●** |  |
| Apo lipoprotein A | **󠄃** |  |  |  |  |  |  |  |  |  |  |  |  |  |  | **●** |
| Apo lipoprotein B | **󠄃** |  |  |  |  |  |  |  |  |  |  |  |  |  |  | **●** |

#### Kidney / Urogenital

| **Items screened** | **Relevance** | | | | | | | | | | **Frequency** | | | | | |
| --- | --- | --- | --- | --- | --- | --- | --- | --- | --- | --- | --- | --- | --- | --- | --- | --- |
|  | 0 | 1 | 2 | 3 | 4 | 5 | 6 | 7 | 8 | 9 | 3-4m | 6m | 12m | 2-5y | Gen | never |
| Risk scores D. A. D. | **󠄃** |  |  |  |  |  |  |  |  |  |  |  |  |  |  | **●** |
| Urine dipstick |  |  |  |  |  |  |  |  | **󠄃** |  |  |  | **●** |  |  |  |
| Pat.grp: <25 |  |  |  |  |  |  |  | **󠄃** |  |  |  |  | **●** |  |  |  |
| Albumin-to-creatinine ratio (ACR) |  |  |  |  |  |  | **󠄃** |  |  |  |  |  | **●** |  |  |  |
| Pat.grp: >50 |  |  |  |  |  |  |  | **󠄃** |  |  |  |  | **●** |  |  |  |
| Pat.grp: Below 200 CD4 |  |  |  |  |  |  |  | **󠄃** |  |  |  |  | **●** |  |  |  |
| Pat.grp: Heterosexual |  |  |  |  |  |  |  | **󠄃** |  |  |  |  | **●** |  |  |  |
| Pat.grp: HP-Country |  |  |  |  |  |  |  | **󠄃** |  |  |  |  | **●** |  |  |  |
| Pat.grp: IVDU |  |  |  |  |  |  |  | **󠄃** |  |  |  |  | **●** |  |  |  |
| Pat.grp: MSM |  |  |  |  |  |  |  | **󠄃** |  |  |  |  | **●** |  |  |  |
| Creatinine |  |  |  |  |  |  |  |  |  | **󠄃** |  | **●** |  |  |  |  |
| Protein-to-creatinine ratio (PCR) |  |  |  |  |  |  |  | **󠄃** |  |  |  |  | **●** |  |  |  |
| Estimated glomerular filtration rate (GFR): (Modification of diet in renal disease equation and Chronic kidney disease (CKD) epi) |  |  |  |  |  |  |  |  | **󠄃** |  |  | **●** |  |  |  |  |
| Estimated glomerular filtration rate (GFR): Chronic kidney disease (CKD) epi |  |  |  |  |  |  |  |  | **󠄃** |  |  | **●** |  |  |  |  |
| Estimated creatinine clearance: COCKROFT GAULT |  |  |  |  |  |  |  |  | **󠄃** |  |  |  |  |  |  | **●** |
| Potassium |  |  |  |  |  |  |  |  | **󠄃** |  |  | **●** |  |  |  |  |
| Pat.grp: <25 |  |  |  |  |  |  |  | **󠄃** |  |  |  | **●** |  |  |  |  |
| Pat.grp: 25-50 |  |  |  |  |  |  |  | **󠄃** |  |  |  | **●** |  |  |  |  |
| Pat.grp: Below 200 CD4 |  |  |  |  |  |  |  | **󠄃** |  |  |  | **●** |  |  |  |  |
| Calcium |  |  |  |  |  |  |  | **󠄃** |  |  |  |  | **●** |  |  |  |
| Phosphorus |  |  |  |  |  |  |  | **󠄃** |  |  |  |  | **●** |  |  |  |
| Sodium |  |  |  |  |  |  |  | **󠄃** |  |  |  | **●** |  |  |  |  |
| Creatinine clearance |  |  |  |  |  |  |  |  | **󠄃** |  |  | **●** |  |  |  |  |
| Pat.grp: <25 |  |  |  |  |  |  |  |  | **󠄃** |  |  |  |  |  |  | **●** |
| Measured glomerular filtration rate (GFR) |  |  |  |  |  |  |  | **󠄃** |  |  |  |  |  |  |  | **●** |
| Ultrasound | **󠄃** |  |  |  |  |  |  |  |  |  |  |  |  |  | **●** |  |
| Urine albumin 24h | **󠄃** |  |  |  |  |  |  |  |  |  |  |  |  |  | **●** |  |
| Urine protein 24h | **󠄃** |  |  |  |  |  |  |  |  |  |  |  |  |  | **●** | **●** |
| Color-Doppler Ultrasound | **󠄃** |  |  |  |  |  |  |  |  |  |  |  |  |  |  | **●** |

#### Liver

| **Items screened** | **Relevance** | | | | | | | | | | **Frequency** | | | | | |
| --- | --- | --- | --- | --- | --- | --- | --- | --- | --- | --- | --- | --- | --- | --- | --- | --- |
|  | 0 | 1 | 2 | 3 | 4 | 5 | 6 | 7 | 8 | 9 | 3-4m | 6m | 12m | 2-5y | Gen | never |
| Alanine Transaminase (ALT) / Aspartate Transaminase (AST) |  |  |  |  |  |  |  |  |  | **󠄃** |  | **●** |  |  |  |  |
| Gamma-glutamyl transferase (GGT), Total bilirubin, Alkaline phosphatase (ALP) |  |  |  |  |  |  |  |  | **󠄃** |  |  | **●** |  |  |  |  |
| Total bilirubin |  |  |  |  |  |  |  |  |  | **󠄃** |  | **●** |  |  |  |  |
| Alkaline phosphatase (ALP) |  |  |  |  |  |  |  |  |  | **󠄃** |  | **●** |  |  |  |  |
| Clotting screen |  |  |  |  |  |  | **󠄃** |  |  |  |  |  |  |  |  | **●** |
| Platelets |  |  |  |  |  |  |  |  | **󠄃** |  |  |  |  |  |  | **●** |
| Hepatitis A B C (D, in B+) screening |  |  |  |  |  |  |  |  |  | **󠄃** |  |  | **●** |  |  |  |
| Non-invasive fibrosis assessment and Elastography (fibroscan) |  |  |  |  |  |  | **󠄃** |  |  |  |  |  |  |  | **●** |  |
| Pat.grp: IVDU |  |  |  |  |  |  |  | **󠄃** |  |  |  |  |  |  | **●** |  |
| Elastography (fibroscan) | **󠄃** |  |  |  |  |  |  |  |  |  |  |  |  |  | **●** |  |
| Ultrasound (US) |  |  |  |  |  |  |  | **󠄃** |  |  |  |  |  |  | **●** |  |
| Pat.grp: <25 |  |  |  |  |  |  | **󠄃** |  |  |  |  |  |  |  | **●** |  |

#### Bone / osteoporosis

| **Items screened** | **Relevance** | | | | | | | | | | **Frequency** | | | | | |
| --- | --- | --- | --- | --- | --- | --- | --- | --- | --- | --- | --- | --- | --- | --- | --- | --- |
|  | 0 | 1 | 2 | 3 | 4 | 5 | 6 | 7 | 8 | 9 | 3-4m | 6m | 12m | 2-5y | Gen | never |
| Fracture risk assessment tool (FRAX) score without DEXA scan |  |  |  |  |  |  | **󠄃** |  |  |  |  |  |  | **●** |  |  |
| Pat.grp: >50 |  |  |  |  |  |  |  |  | **󠄃** |  |  |  | **●** |  |  |  |
| Pat.grp: <25 |  |  |  |  |  | **󠄃** |  |  |  |  |  |  |  |  | **●** |  |
| Pat.grp: Below 200 CD4 |  |  |  |  |  |  |  | **󠄃** |  |  |  |  |  | **●** |  |  |
| Pat.grp: Female |  |  |  |  |  |  |  | **󠄃** |  |  |  |  |  | **●** |  |  |
| Pat.grp: HP-Country (migrants) |  |  |  |  |  |  |  | **󠄃** |  |  |  |  |  | **●** |  |  |
| Fracture risk assessment tool (FRAX) score with DEXA scan |  |  |  |  |  |  | **󠄃** |  |  |  |  |  |  | **●** |  |  |
| Pat.grp: >50 |  |  |  |  |  |  |  |  |  | **󠄃** |  |  | **●** |  |  |  |
| Pat.grp: <25 |  |  |  |  |  | **󠄃** |  |  |  |  |  |  |  | **●** |  |  |
| Pat.grp: Below 200 CD4 |  |  |  |  |  |  |  | **󠄃** |  |  |  |  |  | **●** |  |  |
| Pat.grp: Female |  |  |  |  |  |  |  | **󠄃** |  |  |  |  |  | **●** |  |  |
| Pat.grp: HP-Country |  |  |  |  |  |  |  | **󠄃** |  |  |  |  |  | **●** |  |  |
| Pat.grp: Male |  |  |  |  |  |  |  | **󠄃** |  |  |  |  |  | **●** |  |  |
| 25 OH vitamin D |  |  |  |  |  |  |  | **󠄃** |  |  |  |  | **●** |  |  |  |
| Pat.grp: >50 |  |  |  |  |  |  |  |  | **󠄃** |  |  |  | **●** |  |  |  |
| Bone density |  |  |  |  |  |  |  | **󠄃** |  |  |  |  |  | **●** |  |  |
| Pat.grp: >50 |  |  |  |  |  |  |  |  | **󠄃** |  |  |  |  | **●** |  |  |
| Pat.grp: <25 |  |  |  |  |  | **󠄃** |  |  |  |  |  |  |  |  | **●** |  |
| Pat.grp: 25-50 |  |  |  |  |  |  | **󠄃** |  |  |  |  |  |  | **●** |  |  |
| Pat.grp: Below 200 CD4 |  |  |  |  |  |  | **󠄃** |  |  |  |  |  |  | **●** |  |  |
| Pat.grp: HP-Country |  |  |  |  |  |  |  | **󠄃** |  |  |  |  |  | **●** |  |  |
| Pat.grp: IVDU |  |  |  |  |  |  |  | **󠄃** |  |  |  |  |  | **●** |  |  |
| Hip X-ray | **󠄃** |  |  |  |  |  |  |  |  |  |  |  |  |  |  | **●** |
| Spine X-ray | **󠄃** |  |  |  |  |  |  |  |  |  |  |  |  |  |  | **●** |
| Serum calcium |  |  |  |  |  |  |  | **󠄃** |  |  |  |  | **●** |  |  |  |
| Serum phosphorous |  |  |  |  |  |  |  | **󠄃** |  |  |  |  | **●** |  |  |  |
| Alkaline phosphatase (ALP) |  |  |  |  |  |  |  | **󠄃** |  |  |  | **●** |  |  |  |  |
| Parathyroid hormone (PTH) |  |  |  |  |  | **󠄃** |  |  |  |  |  |  |  |  |  | **●** |
| Pat.grp: >50 |  |  |  |  |  |  | **󠄃** |  |  |  |  |  |  |  |  | **●** |

#### Central and peripheral nervous system

| **Items screened** | **Relevance** | | | | | | | | | | **Frequency** | | | | | |
| --- | --- | --- | --- | --- | --- | --- | --- | --- | --- | --- | --- | --- | --- | --- | --- | --- |
|  | 0 | 1 | 2 | 3 | 4 | 5 | 6 | 7 | 8 | 9 | 3-4m | 6m | 12m | 2-5y | Gen | never |
| Neurocognitiv assessment Test |  |  |  |  |  |  | **󠄃** |  |  |  |  |  |  |  | **●** |  |
| Pat.grp: >50 |  |  |  |  |  |  |  | **󠄃** |  |  |  |  |  | **●** |  |  |
| Fundoscopy | **󠄃** |  |  |  |  |  |  |  |  |  |  |  |  |  | **●** |  |
| Lumbar puncture | **󠄃** |  |  |  |  |  |  |  |  |  |  |  |  |  |  | **●** |
| Computed tomography (CT) | **󠄃** |  |  |  |  |  |  |  |  |  |  |  |  |  | **●** |  |
| Magnetic resonance imaging (MRI) | **󠄃** |  |  |  |  |  |  |  |  |  |  |  |  |  | **●** |  |
| Electroencephalography (EEG) | **󠄃** |  |  |  |  |  |  |  |  |  |  |  |  |  |  | **●** |
| Electromyography | **󠄃** |  |  |  |  |  |  |  |  |  |  |  |  |  |  | **●** |

#### Mental Health

| **Items screened** | **Relevance** | | | | | | | | | | **Frequency** | | | | | |
| --- | --- | --- | --- | --- | --- | --- | --- | --- | --- | --- | --- | --- | --- | --- | --- | --- |
|  | 0 | 1 | 2 | 3 | 4 | 5 | 6 | 7 | 8 | 9 | 3-4m | 6m | 12m | 2-5y | Gen | never |
| Depression / anxiety scale: BECK& HAD |  |  |  |  |  |  |  | **󠄃** |  |  |  |  | **●** |  |  |  |
| Addiction: AUDIT scale (alcohol) | **󠄃** |  |  |  |  |  |  |  |  |  |  |  |  |  |  | **●** |
| Addiction: CAGE scale (alcohol) | **󠄃** |  |  |  |  |  |  |  |  |  |  |  |  |  |  | **●** |
| Addiction: Fagerstrom (tobacco) | **󠄃** |  |  |  |  |  |  |  |  |  |  |  |  |  |  | **●** |
| Addiction: packs per year (tobacco) |  |  |  |  |  |  |  |  | **󠄃** |  |  |  | **●** |  |  |  |
| Addiction to substances |  |  |  |  |  |  |  |  | **󠄃** |  |  |  | **●** |  |  |  |
| Pat.grp: IVDU |  |  |  |  |  |  |  |  |  | **󠄃** |  |  | **●** |  |  |  |
| Other types of addiction (gambling, sex, iPhone…) |  |  |  |  |  |  |  | **󠄃** |  |  |  |  | **●** |  |  |  |
| Pat.grp: IVDU |  |  |  |  |  |  |  |  | **󠄃** |  |  |  | **●** |  |  |  |
| Pat.grp: MSM |  |  |  |  |  |  |  |  | **󠄃** |  |  |  | **●** |  |  |  |
| Pat.grp: Trans |  |  |  |  |  |  |  |  | **󠄃** |  |  |  | **●** |  |  |  |

#### Sexual / reproductive health

| **Items screened** | **Relevance** | | | | | | | | | | **Frequency** | | | | | |
| --- | --- | --- | --- | --- | --- | --- | --- | --- | --- | --- | --- | --- | --- | --- | --- | --- |
|  | 0 | 1 | 2 | 3 | 4 | 5 | 6 | 7 | 8 | 9 | 3-4m | 6m | 12m | 2-5y | Gen | never |
| Syphilis |  |  |  |  |  |  |  |  |  | **󠄃** |  |  | **●** |  |  |  |
| Mycoplasma genitalium | **󠄃** |  |  |  |  |  |  |  |  |  |  |  |  |  |  | **●** |
| Herpes | **󠄃** |  |  |  |  |  |  |  |  |  |  |  |  |  | **●** |  |
| Human papillomavirus (HPV) | **󠄃** |  |  |  |  |  |  |  |  |  |  |  | **●** |  |  |  |
| Viral hepatitis |  |  |  |  |  |  |  |  |  | **󠄃** |  |  | **●** |  |  |  |
| Other sexually transmitted infections (STIs, as Chlamydia, Gonorrhoea, Mycoplasma genitalium, Herpes, and HPV) |  |  |  |  |  |  |  |  | **󠄃** |  |  |  | **●** |  |  |  |
| Pat.grp: MSM |  |  |  |  |  |  |  |  |  | **󠄃** |  |  | **●** |  |  |  |
| Chlamydia PCR (if yes throat, cervical, anal, urethral or urine) |  |  |  |  |  |  |  |  |  | **󠄃** |  |  | **●** |  |  |  |
| Gonorrhoea PCR or / and culture (if yes throat, cervical, anal, urethral or urine) |  |  |  |  |  |  |  |  |  | **󠄃** |  |  | **●** |  |  |  |
| Menopause | **󠄃** |  |  |  |  |  |  |  |  |  |  |  |  |  | **●** |  |
| Assessment contraception (drug interactions) |  |  |  |  |  |  |  |  |  | **󠄃** |  |  | **●** |  |  |  |
| Sexual dysfunction |  |  |  |  |  |  |  | **󠄃** |  |  |  |  | **●** |  |  |  |
| Sexual hormones | **󠄃** |  |  |  |  |  |  |  |  |  |  |  |  |  |  | **●** |

#### Cancer

| **Items screened** | **Relevance** | | | | | | | | | | **Frequency** | | | | | |
| --- | --- | --- | --- | --- | --- | --- | --- | --- | --- | --- | --- | --- | --- | --- | --- | --- |
|  | 0 | 1 | 2 | 3 | 4 | 5 | 6 | 7 | 8 | 9 | 3-4m | 6m | 12m | 2-5y | Gen | never |
| Papanicolaou test (PAP) smear |  |  |  |  |  |  |  |  |  | **󠄃** |  |  | **●** |  |  |  |
| Mammography |  |  |  |  |  |  |  |  | **󠄃** |  |  |  |  |  | **●** |  |
| Chest X-ray |  |  |  |  |  |  |  | **󠄃** |  |  |  |  |  |  | **●** |  |
| Pat.grp: HP-Country |  |  |  |  |  |  |  |  | **󠄃** |  |  |  |  |  | **●** |  |
| Low dose computed tomography (CT) | **󠄃** |  |  |  |  |  |  |  |  |  |  |  |  |  |  | **●** |
| Prostate-specific antigen (PSA) |  |  |  |  |  |  |  | **󠄃** |  |  |  |  |  |  | **●** |  |
| Pat.grp: <25 |  |  |  |  |  | **󠄃** |  |  |  |  |  |  |  |  | **●** |  |
| Pat.grp: 25-50 |  |  |  |  |  | **󠄃** |  |  |  |  |  |  |  |  | **●** |  |
| Anal smear regional Differences? |  |  |  |  |  |  |  | **󠄃** |  |  |  |  |  |  |  | **●** |
| Pat.grp: Female |  |  |  |  |  |  | **󠄃** |  |  |  |  |  |  |  |  | **●** |
| Pat.grp: Heterosexual |  |  |  |  |  |  | **󠄃** |  |  |  |  |  |  |  |  | **●** |
| Pat.grp: HP-Country |  |  |  |  |  |  | **󠄃** |  |  |  |  |  |  |  |  | **●** |
| Pat.grp: IVDU |  |  |  |  |  |  | **󠄃** |  |  |  |  |  |  |  |  | **●** |
| Pat.grp: MSM |  |  |  |  |  |  |  | **󠄃** |  |  |  |  | **●** |  |  |  |
| Pat.grp: Trans |  |  |  |  |  |  |  | **󠄃** |  |  |  |  |  | **●** |  |  |
| Anoscopy |  |  |  |  |  |  | **󠄃** |  |  |  |  |  |  |  |  | **●** |
| Pat.grp: HP-Country |  |  |  |  |  |  | **󠄃** |  |  |  |  |  |  |  | **●** |  |
| Pat.grp: Male |  |  |  |  |  |  |  | **󠄃** |  |  |  |  |  |  |  | **●** |
| Pat.grp: MSM |  |  |  |  |  |  |  |  | **󠄃** |  |  |  | **●** |  |  |  |
| Pat.grp: Trans |  |  |  |  |  |  |  | **󠄃** |  |  |  |  |  |  |  | **●** |
| Do you screen for colon cancer? |  |  |  |  |  |  | **󠄃** |  |  |  |  |  |  |  | **●** |  |
| Pat.grp: >50 |  |  |  |  |  |  |  |  | **󠄃** |  |  |  |  |  | **●** |  |
| Occult blood in faeces | **󠄃** |  |  |  |  |  |  |  |  |  |  |  |  |  | **●** |  |
| Alpha feto protein | **󠄃** |  |  |  |  |  |  |  |  |  |  |  |  |  | **●** |  |
| Liver Ultrasound | **󠄃** |  |  |  |  |  |  |  |  |  |  |  |  |  | **●** |  |

#### Other infectious disease / vaccination

| **Items screened** | **Relevance** | | | | | | | | | | **Frequency** | | | | | |
| --- | --- | --- | --- | --- | --- | --- | --- | --- | --- | --- | --- | --- | --- | --- | --- | --- |
|  | 0 | 1 | 2 | 3 | 4 | 5 | 6 | 7 | 8 | 9 | 3-4m | 6m | 12m | 2-5y | Gen | never |
| Meningococcal vaccination (nat.) |  |  |  |  |  |  | **󠄃** |  |  |  |  |  |  |  | **●** |  |
| Pat.grp: <25 |  |  |  |  |  |  |  | **󠄃** |  |  |  |  |  |  | **●** |  |
| Pat.grp: HP-Country |  |  |  |  |  |  |  | **󠄃** |  |  |  |  |  |  | **●** |  |
| Pat.grp: MSM |  |  |  |  |  |  |  | **󠄃** |  |  |  |  |  |  | **●** |  |
| Hepatitis A vaccination |  |  |  |  |  |  |  |  |  | **󠄃** |  |  |  |  | **●** |  |
| Hepatitis B vaccination |  |  |  |  |  |  |  |  |  | **󠄃** |  |  |  |  | **●** |  |
| Human papilloma virus vaccination |  |  |  |  |  |  |  |  | **󠄃** |  |  |  |  |  | **●** |  |
| Pat.grp: >50 |  |  |  |  |  |  |  | **󠄃** |  |  |  |  |  |  | **●** |  |
| Pat.grp: <25 |  |  |  |  |  |  |  |  |  | **󠄃** |  |  |  |  | **●** |  |
| Influenza vaccination |  |  |  |  |  |  |  |  |  | **󠄃** |  |  | **●** |  |  |  |
| Child born vaccination  (MMR, DTP, VZV) |  |  |  |  |  |  |  |  |  | **󠄃** |  |  |  |  | **●** |  |
| Pat.grp: >50 |  |  |  |  |  |  |  |  | **󠄃** |  |  |  |  |  | **●** |  |
| Pat.grp: Below 200 CD4 |  |  |  |  |  |  |  |  | **󠄃** |  |  |  |  |  | **●** |  |
| Diphtheria, Tetanus and Pertussis (DTP) |  |  |  |  |  |  |  |  |  | **󠄃** |  |  |  |  | **●** |  |
| Varicella-zoster virus (VZV) vaccination | **󠄃** |  |  |  |  |  |  |  |  |  |  |  |  |  | **●** |  |
| Tuberculin skin test (TST) | **󠄃** |  |  |  |  |  |  |  |  |  |  |  |  |  |  | **●** |
| Interferon Gamma Release Assay (IGRA) |  |  |  |  |  |  |  |  | **󠄃** |  |  |  |  |  | **●** |  |
| Pat.grp: HP-Country |  |  |  |  |  |  |  |  |  | **󠄃** |  |  |  |  | **●** |  |
| Toxoplasmosis antibodies |  |  |  |  |  |  |  |  |  | **󠄃** |  |  |  |  | **●** |  |
| Cytomegalovirus (CMV) serology |  |  |  |  |  |  |  |  |  | **󠄃** |  |  |  |  | **●** |  |
| Cytomegalovirus (CMV) polymerase Chain Reaction (PCR) | **󠄃** |  |  |  |  |  |  |  |  |  |  |  |  |  |  | **●** |
| Cryptococcus Antigen | **󠄃** |  |  |  |  |  |  |  |  |  |  |  |  |  |  | **●** |
